# Inference of regulatory networks with a convergence improved MCMC sampler

**DOI:** 10.1186/s12859-015-0734-6

**Published:** 2015-09-24

**Authors:** Nilzair B. Agostinho, Karina S. Machado, Adriano V. Werhli

**Affiliations:** Centro de Ciências Computacionais - C3 Universidade Federal do Rio Grande- FURG, Campus Carreiros, Rio Grande, Brazil

**Keywords:** Bayesian networks, Genetic regulatory networks, Hierarchical bayesian modelling

## Abstract

**Background:**

One of the goals of the Systems Biology community is to have a detailed map of all biological interactions in an organism. One small yet important step in this direction is the creation of biological networks from post-genomic data. Bayesian networks are a very promising model for the inference of regulatory networks in Systems Biology. Usually, Bayesian networks are sampled with a Markov Chain Monte Carlo (MCMC) sampler in the structure space. Unfortunately, conventional MCMC sampling schemes are often slow in mixing and convergence. To improve MCMC convergence, an alternative method is proposed and tested with different sets of data. Moreover, the proposed method is compared with the traditional MCMC sampling scheme.

**Results:**

In the proposed method, a simpler and faster method for the inference of regulatory networks, Graphical Gaussian Models (GGMs), is integrated into the Bayesian network inference, trough a Hierarchical Bayesian model. In this manner, information about the structure obtained from the data with GGMs is taken into account in the MCMC scheme, thus improving mixing and convergence. The proposed method is tested with three types of data, two from simulated models and one from real data. The results are compared with the results of the traditional MCMC sampling scheme in terms of network recovery accuracy and convergence. The results show that when compared with a traditional MCMC scheme, the proposed method presents improved convergence leading to better network reconstruction with less MCMC iterations.

**Conclusions:**

The proposed method is a viable alternative to improve mixing and convergence of traditional MCMC schemes. It allows the use of Bayesian networks with an MCMC sampler with less iterations. The proposed method has always converged earlier than the traditional MCMC scheme. We observe an improvement in accuracy of the recovered networks for the Gaussian simulated data, but this improvement is absent for both real data and data simulated from ODE.

**Electronic supplementary material:**

The online version of this article (doi:10.1186/s12859-015-0734-6) contains supplementary material, which is available to authorized users.

## Background

One of the goals of the Systems Biology community is to have a detailed map of all molecular interactions in an organism. Although much work remains to achieve this goal, the inference of biological networks has become an important tool in Systems Biology. It is now widely recognized that the complexity of organisms is strongly related with the organization of its components in networks. This shifts the interest from the individual behaviour of the components to their orchestrated action. Therefore, the investigation and use of biological networks is highly relevant in the fields of medicine, agriculture, etc. However, these intricate biological networks are for the most part unknown. Owing to the fact that we have at our disposal many different types of measurements taken from the components of these networks one interesting approach would be to try to reconstruct such networks from measurements (data).

In the last few years, several methods for the reconstruction of regulatory networks and biochemical pathways from data have been proposed; see, for instance, [1–4]. For a review of classical methods, see [5–7]. Among various approaches for inferring networks, Bayesian Networks (BNs) are very attractive due to their probabilistic nature and flexibility in incorporating interventions and extra sources of information.

When BNs are adopted as a model for Genetic Regulatory Networks, they are usually sampled in a Markov Chain Monte Carlo (MCMC) scheme. This is because the available data is generally sparse, and it is impossible to enumerate all possible networks even for a reasonable number of nodes. The MCMC scheme has the advantage of being theoretically guaranteed to converge to the posterior distribution [8]. Unfortunately, in practice, MCMC is frequently slow in mixing and convergence and is therefore very computationally expensive. This problem is related with the fact that in the MCMC setup, the movements in the space of networks are based in single edge modifications; thus, the sampler is more easily trapped in local maxima. The concern with MCMC convergence is recurrent and present, e.g., in [9–13].

In [9] the authors address with the problem of convergence and mixing by introducing the proposal moves in the space of node orders. Unfortunately, in this method, the prior probability in network structures cannot be explicitly specified. In [13] the authors propose a new edge reversal move that improves the MCMC convergence when compared with the standard MCMC.

Considering these attempts to solve this problem, in this work we propose an alternative solution that employs a hierarchical Bayesian model to “guide” the MCMC sampling. As the target for this guidance, we use the result from Graphical Gaussian Models (GGMs).

Graphical Gaussian Models (GGMs) are much faster in the task of inferring Genetic Regulatory Networks. The speed of this method comes with a price, however. When compared with BNs, GGMs lack a certain amount of information, as by its nature it cannot either model edge directions or moralize the graph. These features make GGMs inherently less accurate than BNs. Interestingly, GGMs and BNs (scored with the Bayesian Gaussian likelihood equivalent (BGe) metric) share the same underlying statistical model, i.e., the multivariate Gaussian distribution.

Hence, the main aim of this work is to propose a hierarchical Bayesian model that uses the GGM as a “guide” to the MCMC sampling scheme, thus producing better mixing and convergence.

## Methods

### Bayesian networks

A combination of probability theory and graph theory lays the foundations for BNs.

A set of nodes and a set of directed edges define the graphical structure $\mathcal {G}$ of a BN. Nodes represent random variables, and the conditional dependence relationship is represented by edges. The nature of the interactions between nodes and the intensity of these interactions are indicated by the family of conditional probability distributions ${\mathcal F}$ and their parameters **q**, which specify the functional form of the conditional probabilities associated with the edges. The local Markov property, i.e., *A node is conditionally independent of its non descendants given its parents* characterizes a simple and unique rule for expanding the joint probability in terms of simpler conditional probabilities. In accordance with this property, it is mandatory that a BN be a directed acyclic graph (DAG). Consider *X*_1_,*X*_2_,·,*X*_*N*_ to be a set of random variables represented by the nodes *i*∈{1,·,*N*} in the graph. Define $\pi _{i}[\mathcal {G}]$ to be the parents of node *X*_*i*_ in graph $\mathcal {G}$, and let $X_{\pi _{i}[\mathcal {G}]}$ represent the set of random variables associated with $\pi _{i}[\mathcal {G}]$. Then, we can write the expansion for the joint probability as $P(X_{1},\cdot,X_{N}) =\prod _{i=1}^{N} P(X_{i}|X_{\pi _{i}[\mathcal {G}]}) $.

Having at our disposal a set of training data $\mathcal {D}$ the task of learning a BN structure in a score-based approach consists in finding a DAG structure that better explains this data. Note that to learn a BN, it is not necessary to use Bayesian learning; however, in this work, this is the approach applied.

If we define that $\mathbb {G}$ is the space of all models, the first goal is to find a model $\mathcal {G}^{*}\in \mathbb {G}$ that is most supported by the data $\mathcal {D}$, $\mathcal {G}^{*}=\text {argmax}_{\mathcal {G}}\left \{P(\mathcal {G}|\mathcal {D})\right \}$.

If we apply Bayes’ rule, we get $P(\mathcal {G}|\mathcal {D})\propto P(\mathcal {D}|\mathcal {G})P(\mathcal {G})$, where the marginal likelihood implies an integration over the whole parameter space:

(1) $$ P(\mathcal{D}|\mathcal{G})=\int P(\mathcal{D}|\text{\textbf{q}},\mathcal{G})P(\text{\textbf{q}}|\mathcal{G})d\text{\textbf{q}}.   $$

The integral in Eq. (1), our score, is analytically tractable when the data is complete and the prior $P(\text {\textbf {q}}|\mathcal {G})$ and the likelihood $P(\mathcal {D}|\text {\textbf {q}},\mathcal {G})$ satisfy certain regularity conditions [14, 15]. In this work, we employ the scoring metric known as the Bayesian Gaussian likelihood equivalent (BGe) score [16], which assumes that the data come from a multivariate Gaussian distribution.

### MCMC Sampling scheme for BNs

Although there is a method to assign a score to a graphical structure given a data set, the search for high scoring structures is not trivial [17]. The number of structures increases super-exponentially with the number of nodes; thus, it is impossible to list all the structures. Moreover, $P(\mathcal {G}|\mathcal {D})$ will not be properly represented by a single structure $\mathcal {G}^{*}$ when sparse data sets are considered. Hence, an MCMC scheme is adopted [18], which under fairly general regularity conditions is theoretically guaranteed to converge to the posterior distribution [8].

Given a network structure $\mathcal {G}_{\text {old}}$, a new network structure $\mathcal {G}_{\text {new}}$ is proposed from the proposal distribution $Q(\mathcal {G}_{\text {new}}|\mathcal {G}_{\text {old}})$, which is then accepted according to the standard Metropolis-Hastings [8] scheme with the following acceptance probability:

(2) $$ A=\text{min}\left\{\frac{P(\mathcal{D}|\mathcal{G}_{\text{new}})P(\mathcal{G}_{\text{new}})Q(\mathcal{G}_{\text{old}}|\mathcal{G}_{\text{new}})}{P(\mathcal{D}|\mathcal{G}_{\text{old}})P(\mathcal{G}_{\text{old}})Q(\mathcal{G}_{\text{new}}|\mathcal{G}_{\text{old}})},1\right\}  $$

The standard MCMC proposes at each interaction one of the basic operations of adding, removing or reversing an edge. In the following, the standard MCMC scheme of sampling BNs will be called **BN-MCMC**. For more details about this scheme, see [19].

### Graphical gaussian models

Graphical Gaussian models (GGMs) are undirected graphs in which edges represent the partial correlation coefficients. Partial correlation coefficients describe the pairwise correlation between two variables given all the rest of the variables in the domain. In this way, GGMs allow the identification of conditional independence relations among the variables under the assumption of a multivariate Gaussian distribution of the data.

Considering a given data set $\mathcal {D}$, the empirical covariance matrix **C** with elements *C*_*ik*_ is computed and inverted, and the partial correlations *ρ*_*ik*_ are computed from

(3) $$  \rho_{ik} \; = \; - \left(\frac{C^{-1}_{ik}}{\sqrt{C_{ii}^{-1} C_{kk}^{-1}}}\right).  $$

The stable estimation of the covariance matrix and its inverse is the critical step in this method. In [20], the authors proposed a novel covariance matrix estimator regularized by a shrinkage approach that outperforms the previous methods based on bagging [21]. This novel regularized shrinkage covariance estimator is based on the concept of shrinkage and exploits the Ledoit Wolf lemma [22] for analytic calculation of the optimal shrinkage.

An important point to observe when applying GGMs is the following. Consider two variables, *X*_*i*_ and *X*_*k*_. In this case the element *C*_*ik*_ of the covariance matrix **C** is related to the correlation coefficient between these two variables. A high correlation coefficient between these two variables may indicate three distinct types of interaction: direct, indirect, or joint regulation. However, the only interaction of interest for the construction of a network is direct interaction. The strengths of these direct interactions are measured by the partial correlation coefficient *ρ*_*ik*_, which describes the correlation between nodes *X*_*i*_ and *X*_*k*_ conditional on all the other nodes in the network. Thus, partial correlations *ρ*_*ik*_ indicate the strength of the direct interactions, which are the only interactions that have a meaning for the reconstruction of the network.

### BNs guided by GGMs (BNGGM)

When applying MCMC for sampling network structures in a score and search scheme, one of the main problems is the slow mixing and convergence of the MCMC.

In this work, we follow the ideas presented in [23–25] and propose a Hierarchical Bayesian model, hereafter called **BNGGM**, to sample network structures. This allows the MCMC sampling to be “guided” by a faster and coarser method, GGMs, thus improving mixing and convergence of the MCMC. GGMs are said to be coarser than BNs because they are able to represent only the undirected relationships amongst variables, and BNs can represent directed interactions. However, not all interactions are directed in BNs due to the existence of the equivalence classes.

**Fig. 1:**
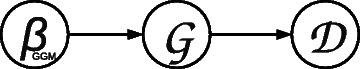
Hierarchical Bayesian model. The probabilistic graphical model represents conditional independence relations between the data $\mathcal {D}$, the network structure $\mathcal {G}$, and the hyperparameter of the prior on GGM, *β*_GGM_. Following the standard rules of factorization in Bayesian networks as defined in [31] we get the expansion: $P(\mathcal {D},\mathcal {G},\beta _{\text {GGM}})= P(\mathcal {D}|\mathcal {G})P(\mathcal {G}|\beta _{\text {GGM}})P(\beta _{\text {GGM}})$

GGM is applied to the data, and information about the graphical structure that gave origin to the data is retrieved. This information is not perfect, but it indicates a potential relationship among the variables. To employ this information together with the MCMC, the probabilistic graphical model presented in Fig. 1 is applied. The probabilistic graphical model represents conditional independence relations between the data $\mathcal {D}$, the network structure $\mathcal {G}$, and the hyperparameter of the prior on GGM, *β*_GGM_.

Moreover, we follow [23] and define the prior distribution over network structures $\mathcal {G}$ to take the form of a Gibbs distribution:

(4) $$  P(\mathcal{G}|\beta_{\text{GGM}})= \frac{e^{-\beta_{\text{GGM}} E(\mathcal{G})}}{Z(\beta_{\text{GGM}})}  $$

where *β*_GGM_ is the hyperparameter of the prior on GGM, *Z*(*β*_GGM_) is a normalizing constant usually referred to as a partition function: $Z(\beta _{\text {GGM}})= \sum _{\mathcal {G}\in \mathbb {G}}e^{-\beta _{\text {GGM}} E(\mathcal {G})}$ and $E(\mathcal {G})$ is the energy of a network $\mathcal {G}$.

The hyperparameter *β*_GGM_ corresponds to an inverse temperature in statistical physics. It can be interpreted as a factor that indicates the strength of the influence of the GGM relative to the data. For *β*_GGM_→0, the prior distribution defined in Eq. (4) becomes flat and uninformative about the network structure. Conversely, for *β*_GGM_→*∞*, the prior distribution becomes sharply peaked at the network structure with the lowest energy.

The energy of a network is defined to be a measure of how similar two networks are. In the present work we are interested in sampling networks that are similar to the networks “suggested” by the GGM model; thus, the definition of energy is as follows.

A network $\mathcal {G}$ can be represented by an adjacency matrix. In this matrix an entry *g*_*ik*_ can assume either the values 0 or 1 representing, respectively, the absence or presence of an edge between nodes *i* and *k*. Additionally, the result of the GGM inference is a matrix, ***ρ***, of partial correlation coefficients *ρ*_*ik*_ where $\{\rho _{\textit {ik}}\in \mathbb {R} |-1\leq \rho _{\textit {ik}}\leq 1\}$. Because our interest lies in the partial correlation strength and not in its value we rescale the matrix ***ρ*** in a manner that its highest and lowest values match, respectively, the presence and the absence of an edge in the adjacency matrix. Each element of the rescaled partial correlation matrix, ***τ***, is obtained by:

(5) $$ \tau_{ik}=\frac{|\rho_{ik}|-\min(|\boldsymbol\rho|)}{\max(|\boldsymbol\rho|)-\min(|\boldsymbol\rho|)}  $$

where |.| represents the absolute value of a number or the element-wise absolute values in a matrix. Note that $\{\tau _{\textit {ik}}\in \mathbb {R} |0\leq \tau _{\textit {ik}}\leq 1\}$.

Having a transformed matrix of correlation coefficients, ***τ***, we consider its entries *τ*_*i*,*k*_ to represent the knowledge about the interactions between nodes as follows:

- If entry *τ*_*i*,*k*_=0.5, it does not provide any knowledge about the presence or absence of the directed edge between nodes *i* and *k*.
- If 0≤*τ*_*i*,*k*_<0.5, it provides evidence that there is no directed edge between nodes *i* and *k*. The evidence is stronger as *τ*_*i*,*k*_ is closer to 0.
- If 0.5<*τ*_*i*,*k*_≤1, we have prior evidence that there is a directed edge pointing from node *i* to node *k*. The evidence is stronger as *τ*_*i*,*k*_ is closer to 1.

Additionally, we define the energy of a network $\mathcal {G}$ as:

(6) $$ E(\mathcal{G})=\sum_{i,k=1}^{N}\left|\tau_{i,k}-g_{i,k}\right|   $$

where *N* is the number of nodes in the network. The more similar the networks $\mathcal {G}$ and ***τ*** are, the lower is the energy *E*. Increasing differences amongst $\mathcal {G}$ and ***τ*** produce higher values of *E*.

### MCMC sampling scheme for BNGGM

Having defined the prior probability distribution over network structures in the previous section, we now define an MCMC sampling scheme to sample from the posterior distribution both the network structure and the hyperparameter.

The goal is to sample the network structure $\mathcal {G}$ and the hyperparameter *β*_GGM_ from the posterior distribution $P\left (\mathcal {G},\beta _{\text {GGM}}|\mathcal {D}\right)$ so that a new network structure $\left (\mathcal {G}'\right)$ and a new hyperparameter (*β*GGM′) are proposed, respectively, from the proposal distributions $Q\left (\mathcal {G}'|\mathcal {G}\right)$ and *R*(*β*GGM′|*β*_GGM_). We then accept this move according to the standard Metropolis-Hastings update rule [8] with the following acceptance probability:

(7) $$ A=\text{min}\left\{\frac{P(\mathcal{D},\mathcal{G}',\beta_{\text{GGM}}')Q(\mathcal{G}|\mathcal{G}')R(\beta_{\text{GGM}}|\beta_{\text{GGM}}')}{P(\mathcal{D},\mathcal{G},\beta_{\text{GGM}})Q(\mathcal{G}'|\mathcal{G})R(\beta_{\text{GGM}}'|\beta_{\text{GGM}})},1\right\}  $$

which due to the conditional independence relationship depicted in Fig. 1 can be expanded as follows:

(8) $$ {\fontsize{8.5pt}{12pt}\selectfont{\begin{aligned} A\,=\,\text{min}\!\left\{\frac{P(\mathcal{D}|\mathcal{G}')P(\mathcal{G}'|\beta_{\text{GGM}}')P(\beta_{\text{GGM}}')Q(\mathcal{G}|\mathcal{G}')R(\beta_{\text{GGM}}|\beta_{\text{GGM}}')}{P(\mathcal{D}|\mathcal{G})P(\mathcal{G}|\beta_{\text{GGM}})P(\beta_{\text{GGM}})Q(\mathcal{G}'|\mathcal{G})R(\beta_{\text{GGM}}'|\beta_{\text{GGM}})},1\right\} \end{aligned}}}  $$

The sampling of both structure and hyperparameter in the same move proposal is likely to produce low acceptance probability. Therefore, we split the move proposal into two sub-moves.

First, we sample a new network structure $\mathcal {G}'$ from the proposal distribution $Q(\mathcal {G}'|\mathcal {G})$ while keeping the hyperparameter *β*_GGM_ fixed, and accept this move with the following acceptance probability:

(9) $$ A(\mathcal{G}'|\mathcal{G})=\text{min}\left\{\frac{P(\mathcal{D}|\mathcal{G}')P(\mathcal{G}'|\beta_{\text{GGM}})Q(\mathcal{G}|\mathcal{G}')}{P(\mathcal{D}|\mathcal{G})P(\mathcal{G}|\beta_{\text{GGM}})Q(\mathcal{G}'|\mathcal{G})},1\right\}  $$

Next, we sample a new hyperparameter *β*_GGM_ from the proposal distribution *R*(*β*GGM′|*β*_GGM_) for a fixed network structure $\mathcal {G}$, and accept this move with the following acceptance probability:

(10) $$ {\fontsize{8.6pt}{12pt}\selectfont{\begin{aligned} A(\beta_{\text{GGM}}'|\beta_{\text{GGM}})=\text{min}\left\{\frac{P(\mathcal{G}|\beta_{\text{GGM}}')P(\beta_{\text{GGM}}')R(\beta_{\text{GGM}}|\beta_{\text{GGM}}')}{P(\mathcal{G}|\beta_{\text{GGM}})P(\beta_{\text{GGM}})R(\beta_{\text{GGM}}'|\beta_{\text{GGM}})},1\right\} \end{aligned}}}  $$

For a uniform prior distribution *P*(*β*_GGM_) and a symmetric proposal distribution *R*(*β*GGM′|*β*_GGM_), this expression simplifies to:

(11) $$ A(\beta_{\text{GGM}}'|\beta_{\text{GGM}})=\text{min}\left\{\frac{P(\mathcal{G}|\beta_{\text{GGM}}')}{P(\mathcal{G}|\beta_{\text{GGM}})},1\right\}.  $$

The two submoves are iterated until some convergence criterion is satisfied. The acceptance probability Eq. (11) can be rewritten as:

(12) $$ A(\beta_{\text{GGM}}'|\beta_{\text{GGM}})=\text{min}\left\{ \frac{e^{-E(\mathcal{G})(\beta_{\text{GGM}}'-\beta_{\text{GGM}})}Z(\beta_{\text{GGM}})}{Z(\beta_{\text{GGM}}')},1\right\}  $$

This equation shows the dependency of the acceptance probability on the partition functions *Z*(*β*_GGM_) and *Z*(*β*GGM′). The calculation of the partition functions implies a summation over the whole space of network structures, which owing to its super-exponential complexity is impractical to obtain. However, considering that all possible networks are valid, we can reduce this complexity to polynomial, thus making it possible to obtain an upper bound on the true partition function. For a detailed discussion about this subject, see [25, 26].

In this section, we presented the usual BN as a model for representing regulatory networks and how to sample BNs in a score and search scheme using an MCMC approach. Moreover, we presented the GGM method which is used in our proposed method. We have then introduced the proposed hierarchical Bayesian model, BNGGM, and its sampling scheme. The proposed method combines a simpler method, GGM, with the BN model to improve the mixing and convergence of the MCMC sampling scheme. In the Results section we compare the MCMC sampling of BNs with the sampling of BNGGMs to verify the improvement in mixing and convergence.

## Simulations

### Data

Three sets of data of a different nature are used to evaluate the proposed method in comparison with the traditional MCMC; they are the following: (i) data generated from a Multivariate Gaussian distribution, (ii) data generated with the GeneNetWeaver tool and (iii) real data from flow Cytometry experiments. Regarding the ability of the methods to learn the network structure from the data, the first type of data should be the easiest because it shares the same underlying model with the learning method, i.e., the Multivariate Gaussian distribution. The second type of data is obtained from a stochastic system of coupled differential equations and is more realistic, making it more difficult for a network to be accurately devised. The real data does not come from a model; hence, it should be the most difficult type of data to infer a network from.

#### Gaussian multivariate data

A clear and simple way of generating synthetic data from a given structure is to sample it from a linear-Gaussian distribution. The random variable *X*_*i*_ denoting the expression of node *i* is distributed according to $X_{i} \; \sim \;N\left (\sum _{k} w_{\textit {ik}}x_{k},\sigma ^{2}\right)$, where *N*(.) denotes the Normal distribution, the sum extends over all parents of node *i*, and *x*_*k*_ represents the value of node *k*. The interaction strength between nodes *X*_*i*_ and *X*_*k*_ is *w*_*ik*_≠0. If *w*_*ik*_=0, node *X*_*k*_ is not a parent of node *X*_*i*_. The value of *σ* ^2^ can be interpreted as being dynamic noise. Low values of *σ* ^2^ indicate a very deterministic data set; conversely, high values of *σ* ^2^ indicate a noisy data set. This process is the equivalent of sampling from a multivariate Gaussian distribution and, hence, a perfect match for the scoring method BGe. The data generated with this method will be referred to in this work as Gaussian data. To generate Gaussian data, we set *w*_*ik*_=1 if the edge is present in the network and *w*_*ik*_=0 otherwise. We also set *σ* ^2^=0.01. These values are based on the work of [25, 27].

#### GeneNetWeaver data

To have more realistic simulated data we use the tool GeneNetWeaver (GNW) [28]. Data generated using GNW is obtained from a stochastic system of coupled differential equations (ODEs) with added noise. This type of data is supposed to be more similar to real data as it presents non-linearities which are typical of real biological systems. However, we are sure about what network structure the inference algorithm should find because the data is simulated from a known structure. Regarding the parameters in the GNW tool, we selected experiments to be “*multifactorial*” with “*add Gaussian nois*e” and “*std dev* =0.005”. Data generated with this method will henceforth be referred to as GNW.

**Fig. 2:**
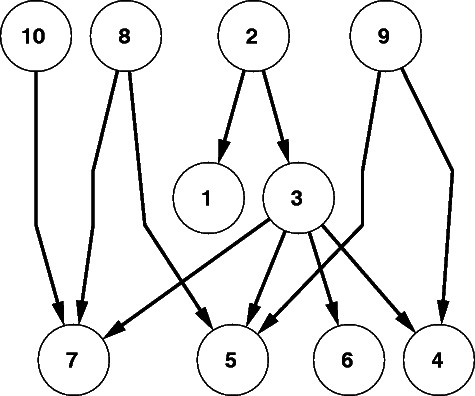
Sub-network *Escherichia Coli*. The graph shows a sub-network extracted from *Escherichia Coli* network. This sub-network is part of the DREAM challenge 3 as presented in [28]

For both types of simulated data, Gaussian and GNW, we obtained data sets from the structure presented in Fig. 2.

#### Real flow-cytometry data

**Fig. 3:**
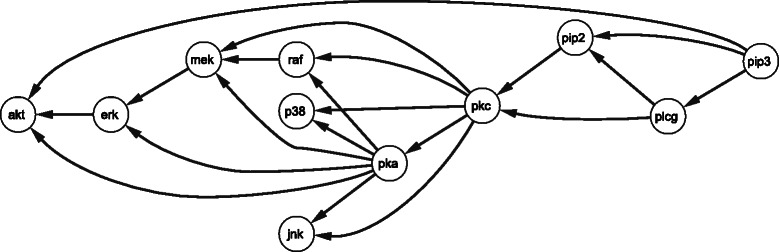
Raf signalling pathway. The graph shows the currently accepted signalling network, adapted from [29]. Nodes represent proteins, edges represent interactions, and arrows indicate the direction of signal transduction

In [29] the authors used intracellular multicolour flow cytometry experiments to measure the concentration levels of the 11 proteins that compose the network depicted in Fig. 3.

This pathway has been extensively studied in the literature (e.g., [29, 30]), hence,

an accepted gold standard network obtained from various distinct studies is available; see Fig. 3. The data produced with this method is regarded as Real data in this work.

The Real data sets are achieved from the structure presented in Fig. 3.

For each one of the three types of data, Gaussian, GNW and Real, we generated five data sets with 100 measurements (data points) each. The GNW and Real data sets were preprocessed before being analysed. We used quantile-normalisation to normalise each of the five data sets. That is, for each of the variables we replaced the 100 measured values by quantiles of the standard normal distribution *N*(0,1). More precisely, for each of the variables the *j*-th highest measured value was replaced by the $\left (\frac {j}{100}\right)$-quantile of the standard normal distribution, whereby the ranks of identical measured values were averaged.

### Simulation setup

In total, we have at our disposal 15 data sets. They are obtained from the three different types of data, Gaussian, GNW and Real, with five data sets in each type.

For each of the data sets and for each of the inference methods, BN and BNGGM, we executed two MCMC simulations. The number of two MCMC simulations permits our analysis of convergence. In total, we performed 60 MCMC simulations. The number of MCMC steps was set to 10^4^, from which samples were taken in intervals of 10 MCMC steps. The first half of the MCMC steps were discarded as the burn-in phase.

Following [25], we set *P*(*β*_GGM_) to be the uniform distribution in the interval [0,30].

### Evaluation

Our results are evaluated in two main aspects. One aspect that we are interested in is the reconstruction accuracy and the other is the quality of mixing and convergence.

The result of the MCMC simulation is a collection of sampled network structures represented in adjacency matrices. From this collection of matrices, we obtain one average matrix, $\mathcal {R}$, where each entry *r*_*ij*_ indicates the marginal posterior probabilities of the edges. To assess the performance of the methods, it is necessary to compare its results with some known network. We call this known network the true network $\mathcal {T}$, where the entries *t*_*ij*_∈{0,1} indicate the presence and the absence of the connection between nodes *X*_*i*_ and *X*_*j*_.

To compare our resulting network $\mathcal {R}$ with the true network $\mathcal {T}$ we transform it in an adjacency matrix, $\mathcal {A_{R}}(\epsilon)$, by imposing a threshold *ε*. Each entry of the adjacency matrix *a*_*ij*_ is 1 if *r*_*ij*_≥*ε* and 0 otherwise.

Having these two matrices, $\mathcal {T}$ and $\mathcal {A_{R}}(\epsilon)$, we can classify each of the edges into categories. An edge can be classified as true positive (TP), false positive (FP), true negative (TN) or false negative (FN); see Table 1 for a summary.

**Table 1:** Classification of edges

*t*_*ij*_	*r*_*ij*_	Category
0	0	TN
0	1	FP
1	0	FN
1	1	TP

This table shows how an edge is classified according to the values in the true matrix (*t*_*ij*_) and in the adjacency matrix (*a*_*ij*_). An entry that is equal to zero means that the edge from node *X*_*i*_ to node *X*_*j*_ is absent, conversely, an entry that is equal to one means that the edge is present

The receiver operator characteristics (ROC) curve is obtained by varying the threshold *ε* and plotting the relative number of TP edges against the relative number of FP edges for each of the thresholds. As it is impractical to compare the whole ROC curves, we instead use the area under the ROC curve (AUC). The AUC summarizes the results for all the thresholds. A perfect predictor would produce an AUC value of 1. Conversely, a random predictor would produce an AUC value of approximately 0.5. In general, bigger area values represent better predictors.

Due to the existence of the equivalence classes, not all of the edges in an inferred Bayesian network are directed. Therefore, to compute the AUC, we consider an undirected edge as the superposition of two directed edges pointing in opposite directions.

## Results and Discussion

The results are presented with two main aims: verify the reconstruction accuracy and assess the quality of mixing and convergence. The results regarding the hyperparameter *β*_GGM_ are presented in the Additional file 1.

Results are shown for two methods: **(i) BN-MCMC** which is the standard sampling of BNs with the structure MCMC and **(ii) BNGGM** which regards the proposed Hierarchical Bayesian model in which network sampling is guided by GGMs results.

**Fig. 4:**
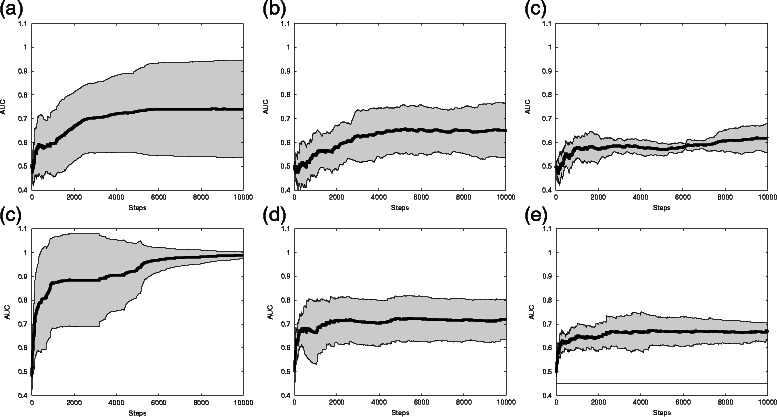
Comparison of AUC values. Each graph presents, for each simulation step, the mean AUC in the inner solid line and one standard deviation in the upper and bottom lines that delimit the shaded gray area. The mean and standard deviation are calculated from five different data sets for each type of data and for the two methods. BN-MCMC results are presented in the top panels, (**a**), (**b**) and (**c**), and the results from BNGGM are presented in the bottom panels, (**d**), (**e**) and (**f**). In left panels, (**a**) and (**d**), are the results from Gaussian data set, the middle panels, (**b**) and (**e**), present the results for GNW data set and the right panel, (**c**) and (**f**), shows results for real flow cytometry data

In Fig. 4, a summary of the results regarding the reconstruction accuracy is presented. To measure the accuracy of reconstruction, we use the AUC (Area Under the ROC Curve), where ROC is the Receiver Operator Characteristics. The vertical axis shows the mean AUC, and the horizontal axis presents the MCMC step. Each graph presents the mean (in the middle line) and standard deviation (in the upper and bottom lines that delimit the shaded grey area) of the AUC calculated from five distinct data sets. The AUC value is calculated for each simulation step; i.e., in each step, we considered this to be the size of the simulation and calculated the AUC value. With this setting, it is possible to analyse what the results would be if the simulation was run for the given number of steps.

The mean and standard deviation are calculated from five different data sets for each type of data and for the two methods. BN-MCMC results are presented in the top panels, (a), (b) and (c), and the results from BNGGM are presented in the bottom panels, (d), (e) and (f). In the left panels, (a) and (d), are the results from the Gaussian data set; middle panels, (b) and (e), present the results from the GNW data set and the right panel, (c) and (f), shows results for real flow cytometry data.

**Fig. 5:**
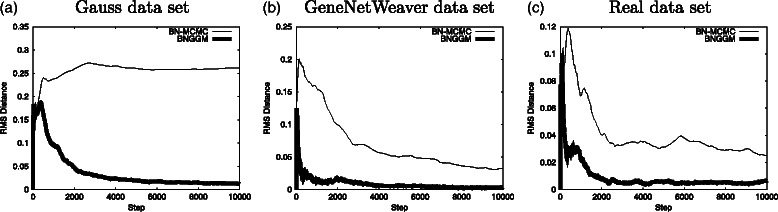
Comparison between the convergence of BN-MCMC and BNGGM methods. Vertical axis presents the *c*_rms_ and horizontal axis shows the MCMC step. In each graph the thin line presents results from BN-MCMC and the thick line presents results from the BNGGM. Each of these lines is the result of running the algorithm twice with the same data set but from different initializations. Panels (**a**), (**b**) and (**c**) present the results for Gaussian, GNW and Real data respectively. The thick line (BNGGM) reaches smaller values faster than the thin line (BN-MCMC) indicating better convergence. Here we show the results for only one data set. For the results of all the remaining data sets please see the Additional file 1

Figure 5 depicts the results regarding the convergence of the MCMC algorithms. To evaluate convergence in an MCMC in which the sampled parameters are graphs (networks), it is usual to run two simulations with different initializations and produce a scatter plot of the posterior probability of the edges. In a long enough simulation, the posterior probabilities of the edges will be very similar, and all the scatter plot points will lie very close to the line *y*=*x*. If simulations have not properly converged, these points are expected to lie far from this line. As a way to verify convergence, one usually inspects these scatter plots and decides if the simulations converged; see, for instance, [9, 13]. The visual verification of convergence only satisfies a necessary condition for convergence and does not guarantee that convergence has been achieved. In this work, we propose a method for measuring the spread of the points around the line *y*=*x* and use this value to evaluate the convergence of the MCMC. We call this measure the convergence rms, or simply *c*_rms_. For an explanation of how we obtain this value, please refer to the Additional file 1.

In Figure 5, typical convergence behaviour is presented for each type of data and for the two inference methods. The vertical axis presents the *c*_rms_, and the horizontal axis shows the MCMC step. In each graph, there are two lines, one presents the results from the BN-MCMC, and the other results are from the BNGGM. Each of these lines is the result of running the algorithm twice from different initializations. Here, we show the graphs for only one data set. The plots for all the data sets are presented in the Additional file 1.

From Fig. 5, it is clear that the new method, BNGGM, converges in less iterations than the BN-MCMC. In the Gaussian data set, left panel, BN-MCMC appears to have not converged even at the end of the entire simulation. In the GNW and Real data sets, both methods appear to have converged. However, the BNGGM presents better convergence at the end of the MCMC and moreover converges earlier than the BN-MCMC. By inspecting these plots, it is possible to observe that with the new method, the simulations for all three types of data could easily have been stopped earlier. In general, it is safe to say that 4×10^3^ steps should have been enough to produce good results.

Despite the indication that the BNGGM has converged in less iterations than BN-MCMC, it is still necessary to check its performance regarding the reconstruction of networks. This verification is necessary because our convergence diagnostics are just a necessary condition for convergence, and it is possible that the simulations have converged to the wrong posterior distribution. If this is the case, the quality of the reconstructed networks should be poor. Figure 4 presents the results in terms of network reconstruction accuracy.

In the Gaussian data set, the difference in both methods is clear. At the end of simulations (10^4^ steps), the BNGGM AUC mean value is very close to 1 and presents very little variance, indicating that the simulations for all data sets have retrieved almost all of the structure of the network correctly. On the other hand, at the same point, BN-MCMC presents very high variance indicating that the simulations have not yet converged. These results are in accordance with the indication in Fig. 5 (a).

The simulations of the GNW data set do not present a significant difference among the methods in the AUC value at the end of the entire simulation. However, it is clear that BNGGM has converged earlier than BN-MCMC and presents lower variance in general, indicating improved convergence.

The results for the Real data set are very similar to those of GNW. This is very interesting and reinforces the notion that the GNW simulated data is similar to the Real data. Again, in this case, we can see that BNGGM appears to have converged earlier than BN-MCMC despite not presenting a significant difference at the end of the whole simulation.

## Conclusions

In this paper, we presented a hierarchical Bayesian model that by combining GGMs with BNs, improves the convergence of the MCMC algorithm applied to the inference of regulatory networks.

If the two methods, BN-MCMC and BNGGM, are run for infinitely long MCMC steps, they will both provide the same result regarding the network reconstruction accuracy. This is expected, as both methods sample networks from the posterior distribution and are guaranteed to converge in an infinitely long simulation. Hence, the principal advantage of the new method is not related with the reconstruction accuracy but instead with the number of MCMC steps necessary to reach convergence. It is clear by inspecting the results that the new method converges earlier than the standard method. Therefore, due to the earlier convergence, it may be possible to run fewer MCMC steps.

When comparing the proposed method with the traditional method, we can observe an interesting feature. When running standard BN-MCMC simulations, it is common for some of these simulations to take a long time to converge, and some simply do not converge in a determined number of simulation steps. Interestingly, when applying the new method, this did not happen in any of the simulations, indicating that the new method “guides” the sampling towards the correct posterior distribution from the beginning of the simulations.

Another attractive aspect of this work is that the extra information used in the BNGGM is obtained from the data itself; thus, there is no need for any other source of data. The extra information is obtained from a distinct method that has the ability to recover such information much faster than the MCMC methodology. It is interesting how the knowledge from the coarser method is transferred to the more refined method. A future research possibility will be to compare the method presented here with the transfer learning methodology. Additionally, we need to investigate the application of the present method in a setting where both methods use different assumptions. For instance, we need to test a coarser method like Mutual Information associated with the multivariate Gaussian model. Because Mutual Information can model non linear interactions, we need to verify if this knowledge can be an advantage in guiding the MCMC sampling.

The main conclusion of this study is that the proposed method improves convergence of MCMC in comparison with the traditional MCMC scheme and, therefore, makes safer the use of MCMC for the sampling of regulatory networks.

## Availability

All the data sets and programs (written in Octave) are available as a zip file in http://tinyurl.com/qh9vf8k. This zip file contains a file named readme.txt that explains how to use the data in conjunction with the programs to reproduce all the results presented in the paper.

## Additional file

Additional file 1 **Supplementary material for the article.** This is a pdf file named bmc_bnggm_supplementary_new.pdf. It can be viewed in any pdf file reader. The file contains an explanation about the score we use in substitution of the visual evaluation for the evaluation of the MCMC convergence. We also put in this supplementary material all the graphs of the results that were the basis for the summarized results presented in the main article. (PDF 1986 kb)
