# Supplementary material for: Inference of regulatory networks with a convergence improved MCMC sampler
Source: BMC Bioinformatics. 2015 Sep 24;16:306. doi: 10.1186/s12859-015-0734-6 (PMC4581096; doi:10.1186/s12859-015-0734-6)
Supplement: Supplementary file 1 — Supplementary material for the article. This is a pdf file named bmc_bnggm_supplementary_new.pdf. It can be viewed in any pdf file reader. The file contains an explanation about the score we use in substitution of the visual evaluation for the evaluation of the MCMC convergence. We also put in this supplementary material all the graphs of the results that were the basis for the summarized results presented in the main article. (PDF 1986 kb) [file 12859_2015_734_MOESM1_ESM.pdf]

RESEARCH

Supplementary material for the article: Inference of regulatory networks with a convergence improved MCMC sampler

Nilzair M Barreto*, Karina S Machado and Adriano V Werhli

*Correspondence:
nilzairmb@gmail.com
Centro de Ciências
Computacionais - C3
Universidade Federal do Rio
Grande - FURG, Campus
Carreiros, Rio Grande, Brazil
Full list of author information is
available at the end of the article
†Equal contributor

Convergence Criterion

Introduction

Markov Chain Monte Carlo (MCMC) is a widespread method for obtaining posterior quantities of interest. It samples successively from the posterior distribution where each sample depends on the previous construction creating in this way the notion of a Markov chain. MCMC has been applied successfully in a variety of problems. However, there are many aspects that have to be addressed when applying such methods. For instance it is possible that the chain does not converge, or that the initial values of parameters have a big influence in the samples. Another possible problem is that the sequence of sampled values show correlation between them.

Markov chain is a powerful tool which is easy to apply and this introduces the risk of serious errors including inappropriate modelling, errors in calculation or programming and slow convergence [1].

Usually to perform diagnosis of convergence graphs or descriptive measures are analysed. These methods are described in [1–7]

Most of these methods are developed for problems in which few parameters, usually between one and three, are sampled in the Markov chain. Problems where convergence diagnostics is performed in samplers that simultaneously sample several parameters are scarce. Hereafter it is described and explained a new method to verify the indication of convergence in the case of high dimensional MCMC samplers.

Heuristic visual evaluation

When sampling a network structure each sample is an adjacency matrix. For a network with n nodes the adjacency matrix has dimension $n \times n$. Thus, in this case, we have to evaluate the convergence of n^2 parameters that are the posterior probabilities over the edges of the network. Because in this setting the number of parameters is usually high the convergence of the MCMC simulations are frequently accessed with a simple heuristic approach. Assuming that the matrix of posterior probabilities is $\mathcal{P}$ each entry p_{ij} of this matrix indicates the marginal posterior probability of the existence of an edge between nodes i and j.

For accessing the MCMC's convergence two simulations are run from different initializations, obtaining two resulting posterior probability matrices, $\mathcal{P}^1$ and $\mathcal{P}^2$. After, a scatter plot is produced by plotting p^1_{ij} against p^2_{ij}, for $1 < i, j < n$.

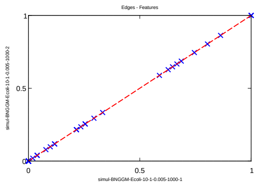

(a) BNGGM - 1000 steps

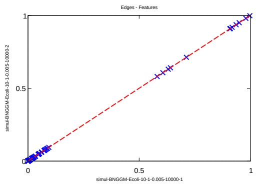

(b) BNGGM - 10000 steps

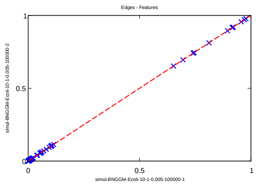

(c) BNGGM - 100000 steps

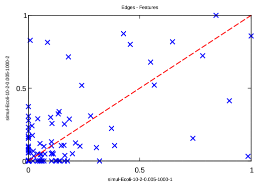

(d) BNMCMC - 1000 steps

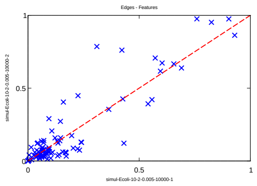

(e) BNMCMC - 10000 steps

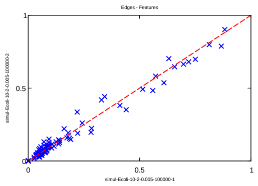

(f) BNMCMC - 100000 steps

Figure 1 Typical examples of the visual heuristic method. The figures in the upper panel show simulations that have converged according to the visual heuristic diagnostic. In the bottom panel the first two figures show simulations that do not have a good indication of convergence, because the values of two simulations are very distinct. The graph to the right in the bottom row shows good indication of convergence. As can be seen from this figure the decision about converge or not is controversial.

In Figure 1 typical scatter plots used for visual convergence diagnosis are shown. The figures in the upper panel show simulations that have converged according to the visual heuristic diagnostic. In the bottom panel the first two figures show simulations that do not have a good indication of convergence, because the values of two simulations are very distinct. The graph to the right in the bottom row shows good indication of convergence. As can be seen from the graphs the decision about convergence from the figures can be controversial. It is interesting to note that this diagnostic of convergence is only a necessary condition for convergence. Therefore, this method does not guarantee the convergence and can only assure the lack of convergence.

Proposal of an score for the visual heuristic evaluation

The method for the diagnosis of convergence proposed in this section is based in the visual heuristic method presented in subsection . Instead of relying on visual analysis we propose to calculate a numeric value that summarizes what is visualized.

Consider that in each step of the MCMC the sampled parameter is a matrix of parameters, $\mathcal{P}$, with dimension $n \times n$. Matrix entries are $p_{ij}, 1 < i, j < n$.

First we calculate de distance of all points to the line $y = x$. The distance, d_{ij}, from a point $P(x_i, y_i)$ to a line with equation $ax + by + c = 0$ is defined by:

$$d_i = \frac{|ax_i + by_i + c|}{\sqrt{(a^2 + b^2)}} \quad (1)$$

Because in our case we are interested in the distance of a point from the identity line, defined by $y = x$, Equation 1 becomes:

$$d_i = \frac{|x_i - y_i|}{\sqrt{2}} \tag{2}$$

We are interested in the spread of the points around the line $y = x$ and we use the rms distance as a measure of this spread. The rms distance is defined by:

$$\text{rms} = \sqrt{\frac{1}{N}\sum_{i}^{N}(d_i)^2} \tag{3}$$

where N is the total number of points and d_i is the distance of the i-th point to the line. If we combine Equation 2 with 3 we get:

$$\text{rms} = \sqrt{\frac{1}{N}\sum_{i}^{N}\frac{|x_i - y_i|^2}{2}} \tag{4}$$

We call this measure the **convergence rms**, or simply c_{rms}.

Supplementary Results

In this section we present more detailed results which have not been included in the main article due to space restrictions.

Hyperparameter β_{GGM}

One important component of the proposed hierarchical Bayesian model is the sampling of the hyperparameter β_{GGM}. For a uniform prior distribution $P(\beta_{\text{GGM}})$ and a symmetric proposal distribution $R(\beta'_{\text{GGM}}|\beta_{\text{GGM}})$, the following acceptance criterion is applied:

$$A(\beta'_{\text{GGM}}|\beta_{\text{GGM}}) = \min\left\{\frac{P(\mathcal{G}|\beta'_{\text{GGM}})}{P(\mathcal{G}|\beta_{\text{GGM}})}, 1\right\} \tag{5}$$

which can be rewritten as:

$$A(\beta'_{\text{GGM}}|\beta_{\text{GGM}}) = \min\left\{\frac{e^{-E(\mathcal{G})(\beta'_{\text{GGM}}-\beta_{\text{GGM}})}Z(\beta_{\text{GGM}})}{Z(\beta'_{\text{GGM}})}, 1\right\} \tag{6}$$

In this work we follow [8] and set $P(\beta_{\text{GGM}})$ to be the uniform distribution in the interval $[0, 30]$. In Figure 2 details of the sampled hyperparameter β_{GGM} are presented for all the data sets in this work. Each column of graphs presents one type of data and each row presents a different data set. Left, centre and right columns presents respectively Gaussian, Ecoli and Real data sets. Each graph presents in the horizontal axis the MCMC step and in the vertical axis the value of β_{GGM}. In [8] it is shown that values of the sampled hyperparameter β_{GGM} close to zero indicate that the prior knowledge is not used by the BN. On the other hand high values of

the sampled hyperparameter indicate that the prior knowledge is used in learning process.

In the present work the sampled values of β_{GGM} in all simulations are greater than zero, indicating that the information obtained from GGM is being used by BNs. This is the expected behaviour because the prior information in the present case comes from the same data which is used in the inference. This use of the information obtained from the GGM leads to the improvement in convergence that we observe in all the results.

AUC diagnostics

One way of indirectly checking convergence is looking to the results in terms of reconstruction accuracy. In order to achieve this we resort to inspecting the values of the AUC for each step in the MCMC simulations. The AUC value is calculated for each simulation step, i.e., in each step we considered that this is the size of the simulation and calculated the AUC value. With this setting it is possible to analyse which would be the results if the simulation was run for the given number of steps. In the main paper we presented the mean and standard deviation of the AUC over the five data sets. Here, in the supplementary material, we show the individual results. In Figure 3 each column of graphs is related to one type of data set. The data set represented in the left, center and right columns are respectively Gaussian, Ecoli and Real. Each row of graphs in Figure 3 is a particular data set. In each graph it is shown the step of the MCMC simulation in the horizontal axis and the value of the AUC from the BNGGM (thick line) and from the BNMCMC (thin line) in the vertical axis.

Convergence criterion

The results considering the convergence criterion presented in Section are shown in Figure 4. In the main paper we presented the result for only one data set and here in the supplementary material we show the results for all the data sets. In Figure 4 each column of graphs is related to one type of data set. The data set represented in the left, center and right columns are respectively Gaussian, Ecoli and Real. Each row of graphs is a particular data set. In each graph the vertical axis presents the c_{rms} and horizontal axis shows the MCMC step. The thin and thick lines in each graph represents respectively BN-MCMC and BNGGM methods. Each of these lines is the result of running the algorithm twice with the same data set but from different initializations.

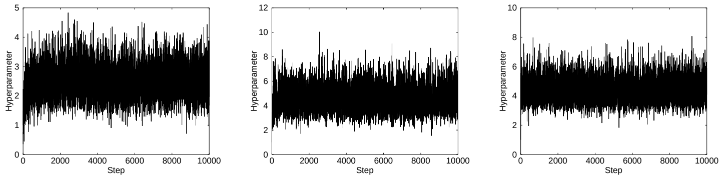

(a) β_{GGM} Gauss data set (b) β_{GGM} Ecoli data set (c) β_{GGM} Real data set

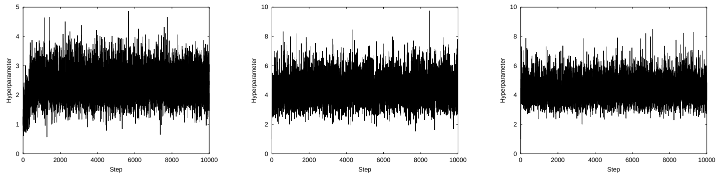

(d) β_{GGM} Gauss data set (e) β_{GGM} Ecoli data set (f) β_{GGM} Real data set

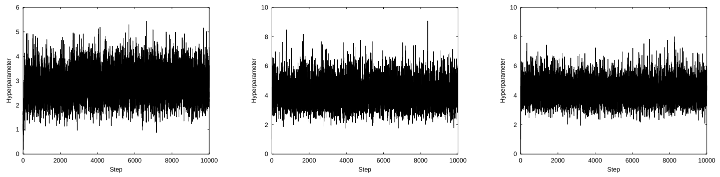

(g) β_{GGM} Gauss data set (h) β_{GGM} Ecoli data set (i) β_{GGM} Real data set

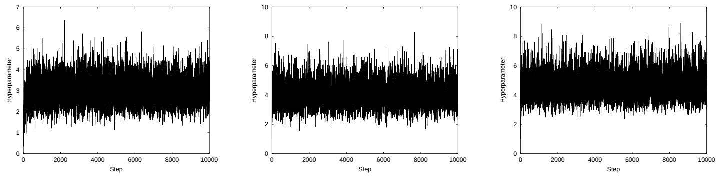

(j) β_{GGM} Gauss data set (k) β_{GGM} Ecoli data set (l) β_{GGM} Real data set

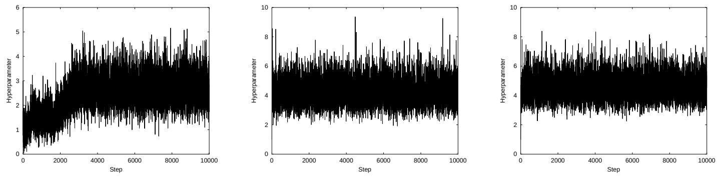

(m) β_{GGM} Gauss data set (n) β_{GGM} Ecoli data set (o) β_{GGM} Real data set

Figure 2 Sampled β_{GGM} for all the data sets. Each column of graphs presents one type of data and each row presents a different data set. Left, centre and right columns presents respectively Gaussian, Ecoli and Real data sets. Each graph presents in the horizontal axis the MCMC step and in the vertical axis the value of β_{GGM}. For all the data sets the sampled values are greater than zero indicating that the information obtained from GGMs are being used in the inference. This supports the findings of improved convergence.

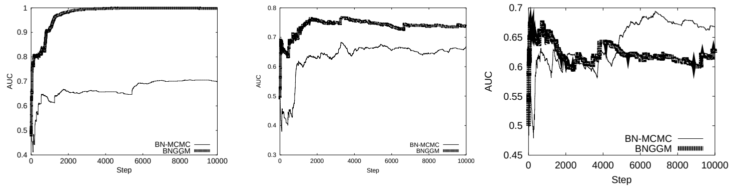

(a) AUC Gauss data set 1 (b) AUC Ecoli data set 1 (c) AUC Real data set 1

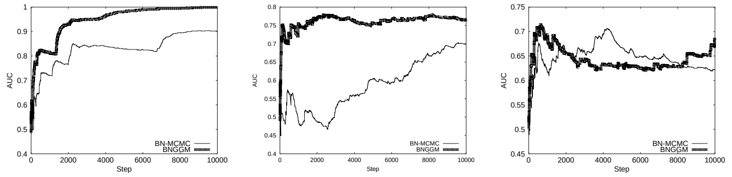

(d) AUC Gauss data set 2 (e) AUC Ecoli data set 2 (f) AUC Real data set 2

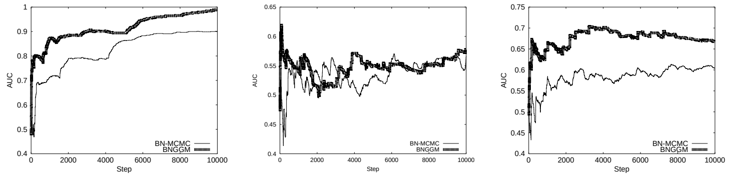

(g) AUC Gauss data set 3 (h) AUC Ecoli data set 3 (i) AUC Real data set 3

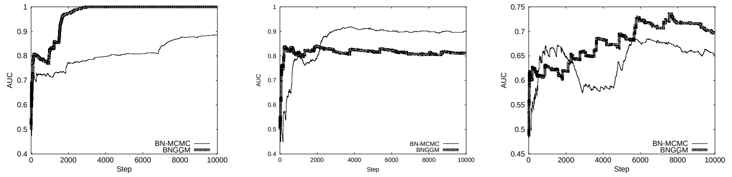

(j) AUC Gauss data set 4 (k) AUC Ecoli data set 4 (l) AUC Real data set 4

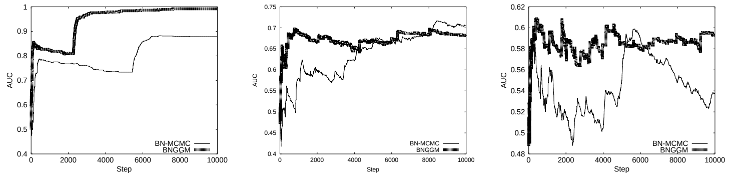

(m) AUC Gauss data set 5 (n) AUC Ecoli data set 5 (o) AUC Real data set 5

Figure 3 AUC values diagnostics. Each column of graphs is related to one type of data set. The data set represented in the left, center and right columns are respectively Gaussian, Ecoli and Real. Each row of graphs in Figure 3 is a particular data set. In each graph it is shown the step of the MCMC simulation in the horizontal axis and the value of the AUC from the BNGGM (thick line) and from the BNMCMC (thin line) in the vertical axis.

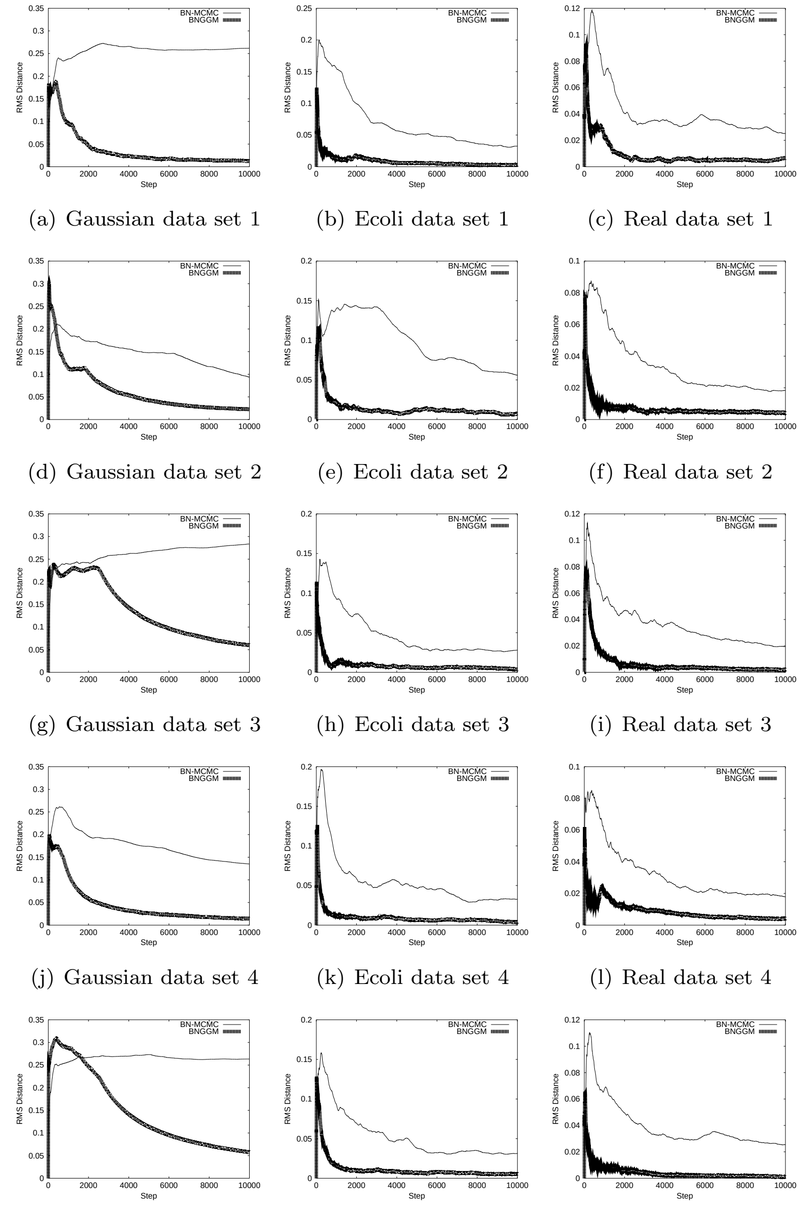

(a) Gaussian data set 1 (b) Ecoli data set 1 (c) Real data set 1

(d) Gaussian data set 2 (e) Ecoli data set 2 (f) Real data set 2

(g) Gaussian data set 3 (h) Ecoli data set 3 (i) Real data set 3

(j) Gaussian data set 4 (k) Ecoli data set 4 (l) Real data set 4

(m) Gaussian data set 5 (n) Ecoli data set 5 (o) Real data set 5

Figure 4 Comparison between the convergence of BN-MCMC and BNGGM methods. The data set represented in the left, center and right columns are respectively Gaussian, Ecoli and Real. Each row of graphs is a particular data set. In each graph the vertical axis presents the c_{rms} and horizontal axis shows the MCMC step. The thin and thick lines in each graph represents respectively BN-MCMC and BNGGM methods. Each of these lines is the result of running the algorithm twice with the same data set but from different initializations.

References

1. Gelman, A., Rubin, D.B.: Inference from iterative simulation using multiple sequence. Statistical Science **4**, 457–472 (1992)
2. Cowles, M.K., Carlin, B.P.: Markov chain monte carlo convergence diagnostics: A comparative review. Journal of the American Statistical Association, **91**, 883–904 (2006)
3. D. Geman, S.G.: Stochastic relaxation, gibbs distributions and the bayesian restoration of images. IEEE Transactions on Pattern Analysis and Machine Intelligence **12**, 609–628 (1984)
4. Gelfand, A.E., Smith, A.F.M.: Sampling-based approaches to calculating marginal densities. Journal of the American Statistical Association **85**, 398–409 (1990)
5. Geweke, J.: Evaluating the accuracy of sampling-based approaches to the calculation of posterior moments. in: Bernardo j m, berger j o, dawid a p, smith a f m (eds.). Bayesian Statistics., 169–193 (1992)
6. J.S. Liu, W.H.W., Kong, A.: Covariance structure of the gibbs sampler with applications to the comparisons of estimators and augmentation schemes. Biometrika **81**, 27–40 (1994)
7. Zellner, A., Min, C.: Gibbs sampler convergence criteria. Journal of the American Statistical Association **90**, 921–927 (1995)
8. Werhli, A.V., Husmeier, D.: Reconstructing gene regulatory networks with Bayesian networks by combining expression data with multiple sources of prior knowledge. Statistical Applications in Genetics and Molecular Biology **6**(1), 15 (2007)
